# Supplementary material for: Resolving the full spectrum of human genome variation using Linked-Reads
Source: Genome Res. 2019 Apr;29(4):635–45. doi: 10.1101/gr.234443.118 (PMC6442396; doi:10.1101/gr.234443.118)
Supplement: Supplemental Material [file supp_gr.234443.118_Supplemental_longranger-2.2.zip › longranger-2.2.tar.bz2!/lr2.2-public/lib/stan/cmdstan/stan/doc/api/html/transform_8hpp_source.html]

Stan: src/stan/prob/transform.hpp Source File


|  |  |
| --- | --- |
|  | Stan  2.5.0  probability, sampling & optimization |


- Main Page
- Related Pages
- Namespaces
- Classes
- Files

- File List
- File Members

All Classes Namespaces Files Functions Variables Typedefs Enumerations Enumerator Friends Macros Pages

- src
- stan
- prob

transform.hpp

Go to the documentation of this file.

1 #ifndef STAN\_\_PROB\_\_TRANSFORM\_HPP

2 #define STAN\_\_PROB\_\_TRANSFORM\_HPP

3

4 #include <cmath>

5 #include <cstddef>

6 #include <limits>

7 #include <stdexcept>

8 #include <sstream>

9 #include <vector>

10

11 #include <boost/throw\_exception.hpp>

12 #include <boost/math/tools/promotion.hpp>

13

14 #include <stan/agrad/rev/matrix.hpp>

15

16 #include <stan/math.hpp>

17 #include <stan/math/error\_handling.hpp>

18 #include <stan/math/error\_handling/matrix/check\_square.hpp>

19 #include <stan/math/matrix.hpp>

20

21 #include <stan/math/matrix/sum.hpp>

22 #include <stan/math/matrix\_error\_handling.hpp>

23 #include <stan/math/matrix/multiply\_lower\_tri\_self\_transpose.hpp>

24 #include <stan/math/matrix/meta/index\_type.hpp>

25

26

27 namespace stan {

28

29  namespace prob {

30

31

32  const double CONSTRAINT\_TOLERANCE = 1E-8;

33

41  template<typename T>

42  void

43  factor\_U(const Eigen::Matrix<T,Eigen::Dynamic,Eigen::Dynamic>& U,

44  Eigen::Array<T,Eigen::Dynamic,1>& CPCs) {

45

46  size\_t K = U.rows();

47  size\_t position = 0;

48  size\_t pull = K - 1;

49

50  if (K == 2) {

51  CPCs(0) = atanh(U(0,1));

52  return;

53  }

54

55  Eigen::Array<T,1,Eigen::Dynamic> temp = U.row(0).tail(pull);

56

57  CPCs.head(pull) = temp;

58

59  Eigen::Array<T,Eigen::Dynamic,1> acc(K);

60  acc(0) = -0.0;

61  acc.tail(pull) = 1.0 - temp.square();

62  for(size\_t i = 1; i < (K - 1); i++) {

63  position += pull;

64  pull--;

65  temp = U.row(i).tail(pull);

66  temp /= sqrt(acc.tail(pull) / acc(i));

67  CPCs.segment(position, pull) = temp;

68  acc.tail(pull) \*= 1.0 - temp.square();

69  }

70  CPCs = 0.5 \* ( (1.0 + CPCs) / (1.0 - CPCs) ).log(); // now unbounded

71  }

72

73

86  template<typename T>

87  bool

88  factor\_cov\_matrix(const Eigen::Matrix<T,Eigen::Dynamic,Eigen::Dynamic>& Sigma,

89  Eigen::Array<T,Eigen::Dynamic,1>& CPCs,

90  Eigen::Array<T,Eigen::Dynamic,1>& sds) {

91

92  size\_t K = sds.rows();

93

94  sds = Sigma.diagonal().array();

95  if( (sds <= 0.0).any() ) return false;

96  sds = sds.sqrt();

97

98  Eigen::DiagonalMatrix<T,Eigen::Dynamic> D(K);

99  D.diagonal() = sds.inverse();

100  sds = sds.log(); // now unbounded

101

102  Eigen::Matrix<T,Eigen::Dynamic,Eigen::Dynamic> R = D \* Sigma \* D;

103  // to hopefully prevent pivoting due to floating point error

104  R.diagonal().setOnes();

105  Eigen::LDLT<Eigen::Matrix<T,Eigen::Dynamic,Eigen::Dynamic> > ldlt;

106  ldlt = R.ldlt();

107  if (!ldlt.isPositive())

108  return false;

109  Eigen::Matrix<T,Eigen::Dynamic,Eigen::Dynamic> U = ldlt.matrixU();

110  factor\_U(U, CPCs);

111  return true;

112  }

113

114  // MATRIX TRANSFORMS +/- JACOBIANS

115

137  template <typename T>

138  Eigen::Matrix<T,Eigen::Dynamic,Eigen::Dynamic>

139  read\_corr\_L(const Eigen::Array<T,Eigen::Dynamic,1>& CPCs, // on (-1,1)

140  const size\_t K) {

141  Eigen::Array<T,Eigen::Dynamic,1> temp;

142  Eigen::Array<T,Eigen::Dynamic,1> acc(K-1);

143  acc.setOnes();

144  // Cholesky factor of correlation matrix

145  Eigen::Array<T,Eigen::Dynamic,Eigen::Dynamic> L(K,K);

146  L.setZero();

147

148  size\_t position = 0;

149  size\_t pull = K - 1;

150

151  L(0,0) = 1.0;

152  L.col(0).tail(pull) = temp = CPCs.head(pull);

153  acc.tail(pull) = 1.0 - temp.square();

154  for(size\_t i = 1; i < (K - 1); i++) {

155  position += pull;

156  pull--;

157  temp = CPCs.segment(position, pull);

158  L(i,i) = sqrt(acc(i-1));

159  L.col(i).tail(pull) = temp \* acc.tail(pull).sqrt();

160  acc.tail(pull) \*= 1.0 - temp.square();

161  }

162  L(K-1,K-1) = sqrt(acc(K-2));

163  return L.matrix();

164  }

165

179  template <typename T>

180  Eigen::Matrix<T,Eigen::Dynamic,Eigen::Dynamic>

181  read\_corr\_matrix(const Eigen::Array<T,Eigen::Dynamic,1>& CPCs,

182  const size\_t K) {

183  Eigen::Matrix<T,Eigen::Dynamic,Eigen::Dynamic> L

184  = read\_corr\_L(CPCs, K);

185  using stan::math::multiply\_lower\_tri\_self\_transpose;

186  return multiply\_lower\_tri\_self\_transpose(L);

187  }

188

213  template <typename T>

214  Eigen::Matrix<T,Eigen::Dynamic,Eigen::Dynamic>

215  read\_corr\_L(const Eigen::Array<T,Eigen::Dynamic,1>& CPCs,

216  const size\_t K,

217  T& log\_prob) {

218

219  using stan::math::log1m;

220  using stan::math::square;

221  using stan::math::sum;

222

223  Eigen::Matrix<T,Eigen::Dynamic,1> values(CPCs.rows() - 1);

224  size\_t pos = 0;

225  // no need to abs() because this Jacobian determinant

226  // is strictly positive (and triangular)

227  // see inverse of Jacobian in equation 11 of LKJ paper

228  for (size\_t k = 1; k <= (K - 2); k++)

229  for (size\_t i = k + 1; i <= K; i++) {

230  values(pos) = (K - k - 1) \* log1m(square(CPCs(pos)));

231  pos++;

232  }

233

234  log\_prob += 0.5 \* sum(values);

235  return read\_corr\_L(CPCs,K);

236  }

237

256  template <typename T>

257  Eigen::Matrix<T,Eigen::Dynamic,Eigen::Dynamic>

258  read\_corr\_matrix(const Eigen::Array<T,Eigen::Dynamic,1>& CPCs,

259  const size\_t K,

260  T& log\_prob) {

261

262  Eigen::Matrix<T,Eigen::Dynamic,Eigen::Dynamic> L

263  = read\_corr\_L(CPCs, K, log\_prob);

264  using stan::math::multiply\_lower\_tri\_self\_transpose;

265  return multiply\_lower\_tri\_self\_transpose(L);

266  }

267

278  template <typename T>

279  Eigen::Matrix<T,Eigen::Dynamic,Eigen::Dynamic>

280  read\_cov\_L(const Eigen::Array<T,Eigen::Dynamic,1>& CPCs,

281  const Eigen::Array<T,Eigen::Dynamic,1>& sds,

282  T& log\_prob) {

283  size\_t K = sds.rows();

284  // adjust due to transformation from correlations to covariances

285  log\_prob += (sds.log().sum() + stan::math::LOG\_2) \* K;

286  return sds.matrix().asDiagonal() \* read\_corr\_L(CPCs, K, log\_prob);

287  }

288

298  template <typename T>

299  Eigen::Matrix<T,Eigen::Dynamic,Eigen::Dynamic>

300  read\_cov\_matrix(const Eigen::Array<T,Eigen::Dynamic,1>& CPCs,

301  const Eigen::Array<T,Eigen::Dynamic,1>& sds,

302  T& log\_prob) {

303

304  Eigen::Matrix<T,Eigen::Dynamic,Eigen::Dynamic> L

305  = read\_cov\_L(CPCs, sds, log\_prob);

306  using stan::math::multiply\_lower\_tri\_self\_transpose;

307  return multiply\_lower\_tri\_self\_transpose(L);

308  }

309

317  template<typename T>

318  Eigen::Matrix<T,Eigen::Dynamic,Eigen::Dynamic>

319  read\_cov\_matrix(const Eigen::Array<T,Eigen::Dynamic,1>& CPCs,

320  const Eigen::Array<T,Eigen::Dynamic,1>& sds) {

321

322  size\_t K = sds.rows();

323  Eigen::DiagonalMatrix<T,Eigen::Dynamic> D(K);

324  D.diagonal() = sds;

325  Eigen::Matrix<T,Eigen::Dynamic,Eigen::Dynamic> L

326  = D \* read\_corr\_L(CPCs, K);

327  using stan::math::multiply\_lower\_tri\_self\_transpose;

328  return multiply\_lower\_tri\_self\_transpose(L);

329  }

330

331

341  template<typename T>

342  const Eigen::Array<T,Eigen::Dynamic,1>

343  make\_nu(const T eta, const size\_t K) {

344  using Eigen::Array;

345  using Eigen::Dynamic;

346  using Eigen::Matrix;

347  using stan::math::index\_type;

348  typedef typename index\_type<Matrix<T,Dynamic,1> >::type size\_type;

349

350  Array<T,Dynamic,1> nu(K \* (K - 1) / 2);

351

352  T alpha = eta + (K - 2.0) / 2.0; // from Lewandowski et. al.

353

354  // Best (1978) implies nu = 2 \* alpha for the dof in a t

355  // distribution that generates a beta variate on (-1,1)

356  T alpha2 = 2.0 \* alpha;

357  for (size\_type j = 0; j < (K - 1); j++) {

358  nu(j) = alpha2;

359  }

360  size\_t counter = K - 1;

361  for (size\_type i = 1; i < (K - 1); i++) {

362  alpha -= 0.5;

363  alpha2 = 2.0 \* alpha;

364  for (size\_type j = i + 1; j < K; j++) {

365  nu(counter) = alpha2;

366  counter++;

367  }

368  }

369  return nu;

370  }

371

372

373

374

375  // IDENTITY

376

389  template <typename T>

390  inline

391  T identity\_constrain(T x) {

392  return x;

393  }

394

408  template <typename T>

409  inline

410  T identity\_constrain(const T x, T& /\*lp\*/) {

411  return x;

412  }

413

425  template <typename T>

426  inline

427  T identity\_free(const T y) {

428  return y;

429  }

430

431

432  // POSITIVE

433

444  template <typename T>

445  inline

446  T positive\_constrain(const T x) {

447  return exp(x);

448  }

449

466  template <typename T>

467  inline

468  T positive\_constrain(const T x, T& lp) {

469  lp += x;

470  return exp(x);

471  }

472

489  template <typename T>

490  inline

491  T positive\_free(const T y) {

492  stan::math::check\_positive("stan::prob::positive\_free(%1%)", y,

493  "Positive variable", (double\*)0);

494  return log(y);

495  }

496

497  // LOWER BOUND

498

518  template <typename T, typename TL>

519  inline

520  T lb\_constrain(const T x, const TL lb) {

521  if (lb == -std::numeric\_limits<double>::infinity())

522  return identity\_constrain(x);

523  return exp(x) + lb;

524  }

525

542  template <typename T, typename TL>

543  inline

544  typename boost::math::tools::promote\_args<T,TL>::type

545  lb\_constrain(const T x, const TL lb, T& lp) {

546  if (lb == -std::numeric\_limits<double>::infinity())

547  return identity\_constrain(x,lp);

548  lp += x;

549  return exp(x) + lb;

550  }

551

567  template <typename T, typename TL>

568  inline

569  typename boost::math::tools::promote\_args<T,TL>::type

570  lb\_free(const T y, const TL lb) {

571  if (lb == -std::numeric\_limits<double>::infinity())

572  return identity\_free(y);

573  stan::math::check\_greater\_or\_equal("stan::prob::lb\_free(%1%)",

574  y, lb, "Lower bounded variable",

575  (double\*)0);

576  return log(y - lb);

577  }

578

579

580  // UPPER BOUND

581

601  template <typename T, typename TU>

602  inline

603  typename boost::math::tools::promote\_args<T,TU>::type

604  ub\_constrain(const T x, const TU ub) {

605  if (ub == std::numeric\_limits<double>::infinity())

606  return identity\_constrain(x);

607  return ub - exp(x);

608  }

609

633  template <typename T, typename TU>

634  inline

635  typename boost::math::tools::promote\_args<T,TU>::type

636  ub\_constrain(const T x, const TU ub, T& lp) {

637  if (ub == std::numeric\_limits<double>::infinity())

638  return identity\_constrain(x,lp);

639  lp += x;

640  return ub - exp(x);

641  }

642

665  template <typename T, typename TU>

666  inline

667  typename boost::math::tools::promote\_args<T,TU>::type

668  ub\_free(const T y, const TU ub) {

669  if (ub == std::numeric\_limits<double>::infinity())

670  return identity\_free(y);

671  stan::math::check\_less\_or\_equal("stan::prob::ub\_free(%1%)",

672  y, ub, "Upper bounded variable",

673  (double\*)0);

674  return log(ub - y);

675  }

676

677

678  // LOWER & UPPER BOUNDS

679

707  template <typename T, typename TL, typename TU>

708  inline

709  typename boost::math::tools::promote\_args<T,TL,TU>::type

710  lub\_constrain(const T x, TL lb, TU ub) {

711  stan::math::check\_less("lub\_constrain(%1%)",lb,ub,"lb",(double\*)0);

712

713  if (lb == -std::numeric\_limits<double>::infinity())

714  return ub\_constrain(x,ub);

715  if (ub == std::numeric\_limits<double>::infinity())

716  return lb\_constrain(x,lb);

717

718  T inv\_logit\_x;

719  if (x > 0) {

720  T exp\_minus\_x = exp(-x);

721  inv\_logit\_x = 1.0 / (1.0 + exp\_minus\_x);

722  // Prevent x from reaching one unless it really really should.

723  if ((x < std::numeric\_limits<double>::infinity())

724  && (inv\_logit\_x == 1))

725  inv\_logit\_x = 1 - 1e-15;

726  } else {

727  T exp\_x = exp(x);

728  inv\_logit\_x = 1.0 - 1.0 / (1.0 + exp\_x);

729  // Prevent x from reaching zero unless it really really should.

730  if ((x > -std::numeric\_limits<double>::infinity())

731  && (inv\_logit\_x== 0))

732  inv\_logit\_x = 1e-15;

733  }

734  return lb + (ub - lb) \* inv\_logit\_x;

735  }

736

778  template <typename T, typename TL, typename TU>

779  typename boost::math::tools::promote\_args<T,TL,TU>::type

780  lub\_constrain(const T x, const TL lb, const TU ub, T& lp) {

781  if (!(lb < ub)) {

782  std::stringstream s;

783  s << "domain error in lub\_constrain; lower bound = " << lb

784  << " must be strictly less than upper bound = " << ub;

785  throw std::domain\_error(s.str());

786  }

787  if (lb == -std::numeric\_limits<double>::infinity())

788  return ub\_constrain(x,ub,lp);

789  if (ub == std::numeric\_limits<double>::infinity())

790  return lb\_constrain(x,lb,lp);

791  T inv\_logit\_x;

792  if (x > 0) {

793  T exp\_minus\_x = exp(-x);

794  inv\_logit\_x = 1.0 / (1.0 + exp\_minus\_x);

795  lp += log(ub - lb) - x - 2 \* log1p(exp\_minus\_x);

796  // Prevent x from reaching one unless it really really should.

797  if ((x < std::numeric\_limits<double>::infinity())

798  && (inv\_logit\_x == 1))

799  inv\_logit\_x = 1 - 1e-15;

800  } else {

801  T exp\_x = exp(x);

802  inv\_logit\_x = 1.0 - 1.0 / (1.0 + exp\_x);

803  lp += log(ub - lb) + x - 2 \* log1p(exp\_x);

804  // Prevent x from reaching zero unless it really really should.

805  if ((x > -std::numeric\_limits<double>::infinity())

806  && (inv\_logit\_x== 0))

807  inv\_logit\_x = 1e-15;

808  }

809  return lb + (ub - lb) \* inv\_logit\_x;

810  }

811

842  template <typename T, typename TL, typename TU>

843  inline

844  typename boost::math::tools::promote\_args<T,TL,TU>::type

845  lub\_free(const T y, TL lb, TU ub) {

846  using stan::math::logit;

847  stan::math::check\_bounded<T, TL, TU, typename scalar\_type<T>::type>

848  ("stan::prob::lub\_free(%1%)",

849  y, lb, ub, "Bounded variable", (double\*)0);

850  if (lb == -std::numeric\_limits<double>::infinity())

851  return ub\_free(y,ub);

852  if (ub == std::numeric\_limits<double>::infinity())

853  return lb\_free(y,lb);

854  return logit((y - lb) / (ub - lb));

855  }

856

857

858  // PROBABILITY

859

873  template <typename T>

874  inline

875  T prob\_constrain(const T x) {

876  using stan::math::inv\_logit;

877  return inv\_logit(x);

878  }

879

901  template <typename T>

902  inline

903  T prob\_constrain(const T x, T& lp) {

904  using stan::math::inv\_logit;

905  using stan::math::log1m;

906  T inv\_logit\_x = inv\_logit(x);

907  lp += log(inv\_logit\_x) + log1m(inv\_logit\_x);

908  return inv\_logit\_x;

909  }

910

925  template <typename T>

926  inline

927  T prob\_free(const T y) {

928  using stan::math::logit;

929  stan::math::check\_bounded<T,double,double,T>

930  ("stan::prob::prob\_free(%1%)",

931  y, 0, 1, "Probability variable",(double\*)0);

932  return logit(y);

933  }

934

935

936  // CORRELATION

937

950  template <typename T>

951  inline

952  T corr\_constrain(const T x) {

953  return tanh(x);

954  }

955

968  template <typename T>

969  inline

970  T corr\_constrain(const T x, T& lp) {

971  using stan::math::log1m;

972  T tanh\_x = tanh(x);

973  lp += log1m(tanh\_x \* tanh\_x);

974  return tanh\_x;

975  }

976

993  template <typename T>

994  inline

995  T corr\_free(const T y) {

996  stan::math::check\_bounded<T,double,double,double>

997  ("stan::prob::lub\_free(%1%)",

998  y, -1, 1, "Correlation variable", (double\*)0);

999  return atanh(y);

1000  }

1001

1002

1003  // Unit vector

1004

1013  template <typename T>

1014  Eigen::Matrix<T,Eigen::Dynamic,1>

1015  unit\_vector\_constrain(const Eigen::Matrix<T,Eigen::Dynamic,1>& y) {

1016  using Eigen::Matrix;

1017  using Eigen::Dynamic;

1018  using stan::math::index\_type;

1019  typedef typename index\_type<Matrix<T,Dynamic,1> >::type size\_type;

1020  int Km1 = y.size();

1021  Matrix<T,Dynamic,1> x(Km1 + 1);

1022  x(0) = 1.0;

1023  const T half\_pi = T(M\_PI/2.0);

1024  for (size\_type k = 1; k <= Km1; ++k) {

1025  T yk\_1 = y(k-1) + half\_pi;

1026  T sin\_yk\_1 = sin(yk\_1);

1027  x(k) = x(k-1)\*sin\_yk\_1;

1028  x(k-1) \*= cos(yk\_1);

1029  }

1030  return x;

1031  }

1032

1042  template <typename T>

1043  Eigen::Matrix<T,Eigen::Dynamic,1>

1044  unit\_vector\_constrain(const Eigen::Matrix<T,Eigen::Dynamic,1>& y, T &lp) {

1045  using Eigen::Matrix;

1046  using Eigen::Dynamic;

1047  using stan::math::index\_type;

1048  typedef typename index\_type<Matrix<T,Dynamic,1> >::type size\_type;

1049

1050  int Km1 = y.size();

1051  Matrix<T,Dynamic,1> x(Km1 + 1);

1052  x(0) = 1.0;

1053  const T half\_pi = T(0.5 \* M\_PI);

1054  for (size\_type k = 1; k <= Km1; ++k) {

1055  T yk\_1 = y(k-1) + half\_pi;

1056  T sin\_yk\_1 = sin(yk\_1);

1057  x(k) = x(k-1) \* sin\_yk\_1;

1058  x(k-1) \*= cos(yk\_1);

1059  if (k < Km1)

1060  lp += (Km1 - k) \* log(fabs(sin\_yk\_1));

1061  }

1062  return x;

1063  }

1064

1065  template <typename T>

1066  Eigen::Matrix<T,Eigen::Dynamic,1>

1067  unit\_vector\_free(const Eigen::Matrix<T,Eigen::Dynamic,1>& x) {

1068  using Eigen::Matrix;

1069  using Eigen::Dynamic;

1070  using stan::math::index\_type;

1071  typedef typename index\_type<Matrix<T,Dynamic,1> >::type size\_type;

1072

1073  stan::math::check\_unit\_vector("stan::prob::unit\_vector\_free(%1%)",

1074  x, "Unit vector variable", (double\*)0);

1075  int Km1 = x.size() - 1;

1076  Matrix<T,Dynamic,1> y(Km1);

1077  T sumSq = x(Km1)\*x(Km1);

1078  const T half\_pi = T(M\_PI/2.0);

1079  for (size\_type k = Km1; --k >= 0; ) {

1080  y(k) = atan2(sqrt(sumSq),x(k)) - half\_pi;

1081  sumSq += x(k)\*x(k);

1082  }

1083  return y;

1084  }

1085

1086

1087  // SIMPLEX

1088

1101  template <typename T>

1102  Eigen::Matrix<T,Eigen::Dynamic,1>

1103  simplex\_constrain(const Eigen::Matrix<T,Eigen::Dynamic,1>& y) {

1104

1105  // cut & paste simplex\_constrain(Eigen::Matrix,T) w/o Jacobian

1106  using Eigen::Matrix;

1107  using Eigen::Dynamic;

1108  using stan::math::index\_type;

1109  using stan::math::inv\_logit;

1110  using stan::math::logit;

1111  using stan::math::log1m;

1112  typedef typename index\_type<Matrix<T,Dynamic,1> >::type size\_type;

1113

1114

1115  int Km1 = y.size();

1116  Matrix<T,Dynamic,1> x(Km1 + 1);

1117  T stick\_len(1.0);

1118  for (size\_type k = 0; k < Km1; ++k) {

1119  T z\_k(inv\_logit(y(k) - log(Km1 - k)));

1120  x(k) = stick\_len \* z\_k;

1121  stick\_len -= x(k);

1122  }

1123  x(Km1) = stick\_len;

1124  return x;

1125  }

1126

1140  template <typename T>

1141  Eigen::Matrix<T,Eigen::Dynamic,1>

1142  simplex\_constrain(const Eigen::Matrix<T,Eigen::Dynamic,1>& y,

1143  T& lp) {

1144

1145  using Eigen::Dynamic;

1146  using Eigen::Matrix;

1147  using stan::math::index\_type;

1148  using stan::math::inv\_logit;

1149  using stan::math::logit;

1150  using stan::math::log1m;

1151  using stan::math::log1p\_exp;

1152

1153  typedef typename index\_type<Matrix<T,Dynamic,1> >::type size\_type;

1154

1155  int Km1 = y.size(); // K = Km1 + 1

1156  Matrix<T,Dynamic,1> x(Km1 + 1);

1157  T stick\_len(1.0);

1158  for (size\_type k = 0; k < Km1; ++k) {

1159  double eq\_share = -log(Km1 - k); // = logit(1.0/(Km1 + 1 - k));

1160  T adj\_y\_k(y(k) + eq\_share);

1161  T z\_k(inv\_logit(adj\_y\_k));

1162  x(k) = stick\_len \* z\_k;

1163  lp += log(stick\_len);

1164  lp -= log1p\_exp(-adj\_y\_k);

1165  lp -= log1p\_exp(adj\_y\_k);

1166  stick\_len -= x(k); // equivalently \*= (1 - z\_k);

1167  }

1168  x(Km1) = stick\_len; // no Jacobian contrib for last dim

1169  return x;

1170  }

1171

1186  template <typename T>

1187  Eigen::Matrix<T,Eigen::Dynamic,1>

1188  simplex\_free(const Eigen::Matrix<T,Eigen::Dynamic,1>& x) {

1189  using Eigen::Dynamic;

1190  using Eigen::Matrix;

1191  using stan::math::index\_type;

1192  using stan::math::logit;

1193

1194  typedef typename index\_type<Matrix<T,Dynamic,1> >::type size\_type;

1195

1196  stan::math::check\_simplex("stan::prob::simplex\_free(%1%)", x, "Simplex variable",

1197  (double\*)0);

1198  int Km1 = x.size() - 1;

1199  Eigen::Matrix<T,Eigen::Dynamic,1> y(Km1);

1200  T stick\_len(x(Km1));

1201  for (size\_type k = Km1; --k >= 0; ) {

1202  stick\_len += x(k);

1203  T z\_k(x(k) / stick\_len);

1204  y(k) = logit(z\_k) + log(Km1 - k);

1205  // note: log(Km1 - k) = logit(1.0 / (Km1 + 1 - k));

1206  }

1207  return y;

1208  }

1209

1210

1211  // ORDERED

1212

1222  template <typename T>

1223  Eigen::Matrix<T,Eigen::Dynamic,1>

1224  ordered\_constrain(const Eigen::Matrix<T,Eigen::Dynamic,1>& x) {

1225  using Eigen::Matrix;

1226  using Eigen::Dynamic;

1227  using stan::math::index\_type;

1228

1229  typedef typename index\_type<Matrix<T,Dynamic,1> >::type size\_type;

1230

1231  size\_type k = x.size();

1232  Matrix<T,Dynamic,1> y(k);

1233  if (k == 0)

1234  return y;

1235  y[0] = x[0];

1236  for (size\_type i = 1; i < k; ++i)

1237  y[i] = y[i-1] + exp(x[i]);

1238  return y;

1239  }

1240

1253  template <typename T>

1254  inline

1255  Eigen::Matrix<T,Eigen::Dynamic,1>

1256  ordered\_constrain(const Eigen::Matrix<T,Eigen::Dynamic,1>& x, T& lp) {

1257  using Eigen::Matrix;

1258  using Eigen::Dynamic;

1259  using stan::math::index\_type;

1260

1261  typedef typename index\_type<Matrix<T,Dynamic,1> >::type size\_type;

1262

1263  for (size\_type i = 1; i < x.size(); ++i)

1264  lp += x(i);

1265  return ordered\_constrain(x);

1266  }

1267

1268

1269

1283  template <typename T>

1284  Eigen::Matrix<T,Eigen::Dynamic,1>

1285  ordered\_free(const Eigen::Matrix<T,Eigen::Dynamic,1>& y) {

1286  stan::math::check\_ordered("stan::prob::ordered\_free(%1%)",

1287  y, "Ordered variable");

1288  using Eigen::Matrix;

1289  using Eigen::Dynamic;

1290  using stan::math::index\_type;

1291  typedef typename index\_type<Matrix<T,Dynamic,1> >::type size\_type;

1292

1293  size\_type k = y.size();

1294  Matrix<T,Dynamic,1> x(k);

1295  if (k == 0)

1296  return x;

1297  x[0] = y[0];

1298  for (size\_type i = 1; i < k; ++i)

1299  x[i] = log(y[i] - y[i-1]);

1300  return x;

1301  }

1302

1303

1304  // POSITIVE ORDERED

1305

1315  template <typename T>

1316  Eigen::Matrix<T,Eigen::Dynamic,1>

1317  positive\_ordered\_constrain(const Eigen::Matrix<T,Eigen::Dynamic,1>& x) {

1318  using Eigen::Matrix;

1319  using Eigen::Dynamic;

1320  using stan::math::index\_type;

1321  typedef typename index\_type<Matrix<T,Dynamic,1> >::type size\_type;

1322

1323  size\_type k = x.size();

1324  Matrix<T,Dynamic,1> y(k);

1325  if (k == 0)

1326  return y;

1327  y[0] = exp(x[0]);

1328  for (size\_type i = 1; i < k; ++i)

1329  y[i] = y[i-1] + exp(x[i]);

1330  return y;

1331  }

1332

1345  template <typename T>

1346  inline

1347  Eigen::Matrix<T,Eigen::Dynamic,1>

1348  positive\_ordered\_constrain(const Eigen::Matrix<T,Eigen::Dynamic,1>& x, T& lp) {

1349  using Eigen::Matrix;

1350  using Eigen::Dynamic;

1351  using stan::math::index\_type;

1352  typedef typename index\_type<Matrix<T,Dynamic,1> >::type size\_type;

1353

1354  for (size\_type i = 0; i < x.size(); ++i)

1355  lp += x(i);

1356  return positive\_ordered\_constrain(x);

1357  }

1358

1359

1360

1374  template <typename T>

1375  Eigen::Matrix<T,Eigen::Dynamic,1>

1376  positive\_ordered\_free(const Eigen::Matrix<T,Eigen::Dynamic,1>& y) {

1377  using Eigen::Matrix;

1378  using Eigen::Dynamic;

1379  using stan::math::index\_type;

1380

1381  typedef typename index\_type<Matrix<T,Dynamic,1> >::type size\_type;

1382

1383  stan::math::check\_positive\_ordered("stan::prob::positive\_ordered\_free(%1%)",

1384  y, "Positive ordered variable", (double\*)0);

1385

1386  size\_type k = y.size();

1387  Matrix<T,Dynamic,1> x(k);

1388  if (k == 0)

1389  return x;

1390  x[0] = log(y[0]);

1391  for (size\_type i = 1; i < k; ++i)

1392  x[i] = log(y[i] - y[i-1]);

1393  return x;

1394  }

1395

1396

1397  // CHOLESKY FACTOR

1398

1410  template <typename T>

1411  Eigen::Matrix<T,Eigen::Dynamic,Eigen::Dynamic>

1412  cholesky\_factor\_constrain(const Eigen::Matrix<T,Eigen::Dynamic,1>& x,

1413  int M,

1414  int N) {

1415  using std::exp;

1416  if (M < N)

1417  throw std::domain\_error("cholesky\_factor\_constrain: num rows must be >= num cols");

1418  if (x.size() != ((N \* (N + 1)) / 2 + (M - N) \* N))

1419  throw std::domain\_error("cholesky\_factor\_constrain: x.size() must"

1420  " be (N \* (N + 1)) / 2 + (M - N) \* N");

1421  Eigen::Matrix<T,Eigen::Dynamic,Eigen::Dynamic> y(M,N);

1422  T zero(0);

1423  int pos = 0;

1424  // upper square

1425  for (int m = 0; m < N; ++m) {

1426  for (int n = 0; n < m; ++n)

1427  y(m,n) = x(pos++);

1428  y(m,m) = exp(x(pos++));

1429  for (int n = m + 1; n < N; ++n)

1430  y(m,n) = zero;

1431  }

1432  // lower rectangle

1433  for (int m = N; m < M; ++m)

1434  for (int n = 0; n < N; ++n)

1435  y(m,n) = x(pos++);

1436  return y;

1437  }

1438

1453  template <typename T>

1454  Eigen::Matrix<T,Eigen::Dynamic,Eigen::Dynamic>

1455  cholesky\_factor\_constrain(const Eigen::Matrix<T,Eigen::Dynamic,1>& x,

1456  int M,

1457  int N,

1458  T& lp) {

1459  // cut-and-paste from above, so checks twice

1460

1461  using stan::math::sum;

1462  if (x.size() != ((N \* (N + 1)) / 2 + (M - N) \* N))

1463  throw std::domain\_error("cholesky\_factor\_constrain: x.size() must be (k choose 2) + k");

1464  int pos = 0;

1465  std::vector<T> log\_jacobians(N);

1466  for (int n = 0; n < N; ++n) {

1467  pos += n;

1468  log\_jacobians[n] = x(pos++);

1469  }

1470  lp += sum(log\_jacobians); // optimized for autodiff vs. direct lp +=

1471  return cholesky\_factor\_constrain(x,M,N);

1472  }

1473

1483  template <typename T>

1484  Eigen::Matrix<T,Eigen::Dynamic,1>

1485  cholesky\_factor\_free(const Eigen::Matrix<T,Eigen::Dynamic,Eigen::Dynamic>& y) {

1486  using std::log;

1487  double result;

1488  if (!stan::math::check\_cholesky\_factor("cholesky\_factor\_free(%1%)",y,"y",&result))

1489  throw std::domain\_error("cholesky\_factor\_free: y is not a Cholesky factor");

1490  int M = y.rows();

1491  int N = y.cols();

1492  Eigen::Matrix<T,Eigen::Dynamic,1> x((N \* (N + 1)) / 2 + (M - N) \* N);

1493  int pos = 0;

1494  // lower triangle of upper square

1495  for (int m = 0; m < N; ++m) {

1496  for (int n = 0; n < m; ++n)

1497  x(pos++) = y(m,n);

1498  // diagonal of upper square

1499  x(pos++) = log(y(m,m));

1500  }

1501  // lower rectangle

1502  for (int m = N; m < M; ++m)

1503  for (int n = 0; n < N; ++n)

1504  x(pos++) = y(m,n);

1505  return x;

1506  }

1507

1508  // CHOLESKY CORRELATION MATRIX

1509

1510  template <typename T>

1511  Eigen::Matrix<T,Eigen::Dynamic,Eigen::Dynamic>

1512  cholesky\_corr\_constrain(const Eigen::Matrix<T,Eigen::Dynamic,1>& y,

1513  int K) {

1514  using std::sqrt;

1515  using Eigen::Matrix;

1516  using Eigen::Dynamic;

1517  using stan::math::square;

1518  int k\_choose\_2 = (K \* (K - 1)) / 2;

1519  if (k\_choose\_2 != y.size()) {

1520  std::cout << "k\_choose\_2 = " << k\_choose\_2 << " y.size()=" << y.size() << std::endl;

1521  throw std::domain\_error("y is not a valid unconstrained cholesky correlation matrix."

1522  "Require (K choose 2) elements in y.");

1523  }

1524  Matrix<T,Dynamic,1> z(k\_choose\_2);

1525  for (int i = 0; i < k\_choose\_2; ++i)

1526  z(i) = corr\_constrain(y(i));

1527  Matrix<T,Dynamic,Dynamic> x(K,K);

1528  if (K == 0) return x;

1529  T zero(0);

1530  for (int j = 1; j < K; ++j)

1531  for (int i = 0; i < j; ++i)

1532  x(i,j) = zero;

1533  x(0,0) = 1;

1534  int k = 0;

1535  for (int i = 1; i < K; ++i) {

1536  x(i,0) = z(k++);

1537  T sum\_sqs(square(x(i,0)));

1538  for (int j = 1; j < i; ++j) {

1539  x(i,j) = z(k++) \* sqrt(1.0 - sum\_sqs);

1540  sum\_sqs += square(x(i,j));

1541  }

1542  x(i,i) = sqrt(1.0 - sum\_sqs);

1543  }

1544  return x;

1545  }

1546

1547  // FIXME to match above after debugged

1548  template <typename T>

1549  Eigen::Matrix<T,Eigen::Dynamic,Eigen::Dynamic>

1550  cholesky\_corr\_constrain(const Eigen::Matrix<T,Eigen::Dynamic,1>& y,

1551  int K,

1552  T& lp) {

1553  using std::sqrt;

1554  using Eigen::Matrix;

1555  using Eigen::Dynamic;

1556  using stan::math::log1m;

1557  using stan::math::square;

1558  int k\_choose\_2 = (K \* (K - 1)) / 2;

1559  if (k\_choose\_2 != y.size()) {

1560  std::cout << "k\_choose\_2 = " << k\_choose\_2 << " y.size()=" << y.size() << std::endl;

1561  throw std::domain\_error("y is not a valid unconstrained cholesky correlation matrix."

1562  " Require (K choose 2) elements in y.");

1563  }

1564  Matrix<T,Dynamic,1> z(k\_choose\_2);

1565  for (int i = 0; i < k\_choose\_2; ++i)

1566  z(i) = corr\_constrain(y(i),lp);

1567  Matrix<T,Dynamic,Dynamic> x(K,K);

1568  if (K == 0) return x;

1569  T zero(0);

1570  for (int j = 1; j < K; ++j)

1571  for (int i = 0; i < j; ++i)

1572  x(i,j) = zero;

1573  x(0,0) = 1;

1574  int k = 0;

1575  for (int i = 1; i < K; ++i) {

1576  x(i,0) = z(k++);

1577  T sum\_sqs = square(x(i,0));

1578  for (int j = 1; j < i; ++j) {

1579  lp += 0.5 \* log1m(sum\_sqs);

1580  x(i,j) = z(k++) \* sqrt(1.0 - sum\_sqs);

1581  sum\_sqs += square(x(i,j));

1582  }

1583  x(i,i) = sqrt(1.0 - sum\_sqs);

1584  }

1585  return x;

1586  }

1587

1588

1589

1590  template <typename T>

1591  Eigen::Matrix<T,Eigen::Dynamic,1>

1592  cholesky\_corr\_free(const Eigen::Matrix<T,Eigen::Dynamic,Eigen::Dynamic>& x) {

1593  using std::sqrt;

1594  using Eigen::Matrix;

1595  using Eigen::Dynamic;

1596  using stan::math::square;

1597

1598  double bad\_result; // won't be used

1599  stan::math::check\_square("cholesky\_corr\_free",x,"x",&bad\_result);

1600  // should validate lower-triangular, unit lengths

1601

1602  int K = (x.rows() \* (x.rows() - 1)) / 2;

1603  Matrix<T,Dynamic,1> z(K);

1604  int k = 0;

1605  for (int i = 1; i < x.rows(); ++i) {

1606  z(k++) = corr\_free(x(i,0));

1607  double sum\_sqs = square(x(i,0));

1608  for (int j = 1; j < i; ++j) {

1609  z(k++) = corr\_free(x(i,j) / sqrt(1.0 - sum\_sqs));

1610  sum\_sqs += square(x(i,j));

1611  }

1612  }

1613  return z;

1614  }

1615

1616  // CORRELATION MATRIX

1617

1642  template <typename T>

1643  Eigen::Matrix<T,Eigen::Dynamic,Eigen::Dynamic>

1644  corr\_matrix\_constrain(const Eigen::Matrix<T,Eigen::Dynamic,1>& x,

1645  typename math::index\_type<Eigen::Matrix<T,Eigen::Dynamic,1> >::type k) {

1646

1647  using Eigen::Dynamic;

1648  using Eigen::Matrix;

1649  using stan::math::index\_type;

1650  typedef typename index\_type<Matrix<T,Dynamic,1> >::type size\_type;

1651

1652  size\_type k\_choose\_2 = (k \* (k - 1)) / 2;

1653  if (k\_choose\_2 != x.size())

1654  throw std::invalid\_argument ("x is not a valid correlation matrix");

1655  Eigen::Array<T,Eigen::Dynamic,1> cpcs(k\_choose\_2);

1656  for (size\_type i = 0; i < k\_choose\_2; ++i)

1657  cpcs[i] = corr\_constrain(x[i]);

1658  return read\_corr\_matrix(cpcs,k);

1659  }

1660

1680  template <typename T>

1681  Eigen::Matrix<T,Eigen::Dynamic,Eigen::Dynamic>

1682  corr\_matrix\_constrain(const Eigen::Matrix<T,Eigen::Dynamic,1>& x,

1683  typename math::index\_type<Eigen::Matrix<T,Eigen::Dynamic,1> >::type k,

1684  T& lp) {

1685  using Eigen::Array;

1686  using Eigen::Dynamic;

1687  using Eigen::Matrix;

1688  using stan::math::index\_type;

1689  typedef typename index\_type<Matrix<T,Dynamic,1> >::type size\_type;

1690

1691  size\_type k\_choose\_2 = (k \* (k - 1)) / 2;

1692  if (k\_choose\_2 != x.size())

1693  throw std::invalid\_argument ("x is not a valid correlation matrix");

1694  Array<T,Dynamic,1> cpcs(k\_choose\_2);

1695  for (size\_type i = 0; i < k\_choose\_2; ++i)

1696  cpcs[i] = corr\_constrain(x[i],lp);

1697  return read\_corr\_matrix(cpcs,k,lp);

1698  }

1699

1720  template <typename T>

1721  Eigen::Matrix<T,Eigen::Dynamic,1>

1722  corr\_matrix\_free(const Eigen::Matrix<T,Eigen::Dynamic,Eigen::Dynamic>& y) {

1723  using Eigen::Array;

1724  using Eigen::Dynamic;

1725  using Eigen::Matrix;

1726  using stan::math::index\_type;

1727  typedef typename index\_type<Matrix<T,Dynamic,1> >::type size\_type;

1728

1729  size\_type k = y.rows();

1730  if (y.cols() != k)

1731  throw std::domain\_error("y is not a square matrix");

1732  if (k == 0)

1733  throw std::domain\_error("y has no elements");

1734  size\_type k\_choose\_2 = (k \* (k-1)) / 2;

1735  Array<T,Dynamic,1> x(k\_choose\_2);

1736  Array<T,Dynamic,1> sds(k);

1737  bool successful = factor\_cov\_matrix(y,x,sds);

1738  if (!successful)

1739  throw std::runtime\_error("factor\_cov\_matrix failed on y");

1740  for (size\_type i = 0; i < k; ++i) {

1741  // sds on log scale unconstrained

1742  if (fabs(sds[i] - 0.0) >= CONSTRAINT\_TOLERANCE) {

1743  std::stringstream s;

1744  s << "all standard deviations must be zero."

1745  << " found log(sd[" << i << "])=" << sds[i] << std::endl;

1746  BOOST\_THROW\_EXCEPTION(std::runtime\_error(s.str()));

1747  }

1748  }

1749  return x.matrix();

1750  }

1751

1752

1753  // COVARIANCE MATRIX

1754

1767  template <typename T>

1768  Eigen::Matrix<T,Eigen::Dynamic,Eigen::Dynamic>

1769  cov\_matrix\_constrain(const Eigen::Matrix<T,Eigen::Dynamic,1>& x,

1770  typename math::index\_type<Eigen::Matrix<T,Eigen::Dynamic,1> >::type K) {

1771  using std::exp;

1772

1773  using Eigen::Dynamic;

1774  using Eigen::Matrix;

1775  using stan::math::index\_type;

1776  using stan::math::multiply\_lower\_tri\_self\_transpose;

1777  typedef typename index\_type<Matrix<T,Dynamic,Dynamic> >::type size\_type;

1778

1779  Matrix<T,Dynamic,Dynamic> L(K,K);

1780  if (x.size() != (K \* (K + 1)) / 2)

1781  throw std::domain\_error("x.size() != K + (K choose 2)");

1782  int i = 0;

1783  for (size\_type m = 0; m < K; ++m) {

1784  for (int n = 0; n < m; ++n)

1785  L(m,n) = x(i++);

1786  L(m,m) = exp(x(i++));

1787  for (size\_type n = m + 1; n < K; ++n)

1788  L(m,n) = 0.0;

1789  }

1790  return multiply\_lower\_tri\_self\_transpose(L);

1791  }

1792

1793

1806  template <typename T>

1807  Eigen::Matrix<T,Eigen::Dynamic,Eigen::Dynamic>

1808  cov\_matrix\_constrain(const Eigen::Matrix<T,Eigen::Dynamic,1>& x,

1809  typename math::index\_type<Eigen::Matrix<T,

1810  Eigen::Dynamic,

1811  Eigen::Dynamic> >::type K,

1812  T& lp) {

1813  using std::exp;

1814

1815  using Eigen::Dynamic;

1816  using Eigen::Matrix;

1817  using stan::math::index\_type;

1818  typedef typename index\_type<Matrix<T,Dynamic,Dynamic> >::type size\_type;

1819

1820  if (x.size() != (K \* (K + 1)) / 2)

1821  throw std::domain\_error("x.size() != K + (K choose 2)");

1822  Matrix<T,Dynamic,Dynamic> L(K,K);

1823  int i = 0;

1824  for (size\_type m = 0; m < K; ++m) {

1825  for (size\_type n = 0; n < m; ++n)

1826  L(m,n) = x(i++);

1827  L(m,m) = exp(x(i++));

1828  for (size\_type n = m + 1; n < K; ++n)

1829  L(m,n) = 0.0;

1830  }

1831  // Jacobian for complete transform, including exp() above

1832  lp += (K \* stan::math::LOG\_2); // needless constant; want propto

1833  for (int k = 0; k < K; ++k)

1834  lp += (K - k + 1) \* log(L(k,k)); // only +1 because index from 0

1835  return L \* L.transpose();

1836  // return tri\_multiply\_transpose(L);

1837  }

1838

1861  template <typename T>

1862  Eigen::Matrix<T,Eigen::Dynamic,1>

1863  cov\_matrix\_free(const Eigen::Matrix<T,Eigen::Dynamic,Eigen::Dynamic>& y) {

1864  using std::log;

1865  int K = y.rows();

1866  if (y.cols() != K)

1867  throw std::domain\_error("y is not a square matrix");

1868  if (K == 0)

1869  throw std::domain\_error("y has no elements");

1870  for (int k = 0; k < K; ++k)

1871  if (!(y(k,k) > 0.0))

1872  throw std::domain\_error("y has non-positive diagonal");

1873  Eigen::Matrix<T,Eigen::Dynamic,1> x((K \* (K + 1)) / 2);

1874  // FIXME: see Eigen LDLT for rank-revealing version -- use that

1875  // even if less efficient?

1876  Eigen::LLT<Eigen::Matrix<T,Eigen::Dynamic,Eigen::Dynamic> >

1877  llt(y.rows());

1878  llt.compute(y);

1879  Eigen::Matrix<T,Eigen::Dynamic,Eigen::Dynamic> L = llt.matrixL();

1880  int i = 0;

1881  for (int m = 0; m < K; ++m) {

1882  for (int n = 0; n < m; ++n)

1883  x(i++) = L(m,n);

1884  x(i++) = log(L(m,m));

1885  }

1886  return x;

1887  }

1888

1908  template <typename T>

1909  Eigen::Matrix<T,Eigen::Dynamic,Eigen::Dynamic>

1910  cov\_matrix\_constrain\_lkj(const Eigen::Matrix<T,Eigen::Dynamic,1>& x,

1911  size\_t k) {

1912  size\_t k\_choose\_2 = (k \* (k - 1)) / 2;

1913  Eigen::Array<T,Eigen::Dynamic,1> cpcs(k\_choose\_2);

1914  int pos = 0;

1915  for (size\_t i = 0; i < k\_choose\_2; ++i)

1916  cpcs[i] = corr\_constrain(x[pos++]);

1917  Eigen::Array<T,Eigen::Dynamic,1> sds(k);

1918  for (size\_t i = 0; i < k; ++i)

1919  sds[i] = positive\_constrain(x[pos++]);

1920  return read\_cov\_matrix(cpcs, sds);

1921  }

1922

1947  template <typename T>

1948  Eigen::Matrix<T,Eigen::Dynamic,Eigen::Dynamic>

1949  cov\_matrix\_constrain\_lkj(const Eigen::Matrix<T,Eigen::Dynamic,1>& x,

1950  size\_t k,

1951  T& lp) {

1952  size\_t k\_choose\_2 = (k \* (k - 1)) / 2;

1953  Eigen::Array<T,Eigen::Dynamic,1> cpcs(k\_choose\_2);

1954  int pos = 0;

1955  for (size\_t i = 0; i < k\_choose\_2; ++i)

1956  cpcs[i] = corr\_constrain(x[pos++], lp);

1957  Eigen::Array<T,Eigen::Dynamic,1> sds(k);

1958  for (size\_t i = 0; i < k; ++i)

1959  sds[i] = positive\_constrain(x[pos++],lp);

1960  return read\_cov\_matrix(cpcs, sds, lp);

1961  }

1962

1981  template <typename T>

1982  Eigen::Matrix<T,Eigen::Dynamic,1>

1983  cov\_matrix\_free\_lkj(

1984  const Eigen::Matrix<T,Eigen::Dynamic,Eigen::Dynamic>& y) {

1985

1986  using Eigen::Array;

1987  using Eigen::Dynamic;

1988  using Eigen::Matrix;

1989  using stan::math::index\_type;

1990  typedef typename index\_type<Matrix<T,Dynamic,Dynamic> >::type size\_type;

1991

1992  size\_type k = y.rows();

1993  if (y.cols() != k)

1994  throw std::domain\_error("y is not a square matrix");

1995  if (k == 0)

1996  throw std::domain\_error("y has no elements");

1997  size\_type k\_choose\_2 = (k \* (k-1)) / 2;

1998  Array<T,Dynamic,1> cpcs(k\_choose\_2);

1999  Array<T,Dynamic,1> sds(k);

2000  bool successful = factor\_cov\_matrix(y,cpcs,sds);

2001  if (!successful)

2002  throw std::runtime\_error ("factor\_cov\_matrix failed on y");

2003  Matrix<T,Dynamic,1> x(k\_choose\_2 + k);

2004  size\_type pos = 0;

2005  for (size\_type i = 0; i < k\_choose\_2; ++i)

2006  x[pos++] = cpcs[i];

2007  for (size\_type i = 0; i < k; ++i)

2008  x[pos++] = sds[i];

2009  return x;

2010  }

2011

2012  }

2013

2014 }

2015

2016 #endif

stan::agrad::log1m

fvar< T > log1m(const fvar< T > &x)

**Definition:** log1m.hpp:16

matrix.hpp

stan::math::square

T square(const T x)

Return the square of the specified argument.

**Definition:** square.hpp:22

stan::math::LOG\_2

const double LOG\_2

The natural logarithm of 2, .

**Definition:** constants.hpp:32

stan::agrad::fabs

fvar< T > fabs(const fvar< T > &x)

**Definition:** fabs.hpp:18

stan::prob::positive\_ordered\_free

Eigen::Matrix< T, Eigen::Dynamic, 1 > positive\_ordered\_free(const Eigen::Matrix< T, Eigen::Dynamic, 1 > &y)

Return the vector of unconstrained scalars that transform to the specified positive ordered vector...

**Definition:** transform.hpp:1376

stan::math::check\_ordered

bool check\_ordered(const char \*function, const Eigen::Matrix< T\_y, Eigen::Dynamic, 1 > &y, const char \*name, T\_result \*result)

Return true if the specified vector is sorted into increasing order.

**Definition:** check\_ordered.hpp:29

stan::agrad::log1p\_exp

fvar< T > log1p\_exp(const fvar< T > &x)

**Definition:** log1p\_exp.hpp:15

stan::math::log1p\_exp

boost::math::tools::promote\_args< T >::type log1p\_exp(const T a)

Calculates the log of 1 plus the exponential of the specified value without overflow.

**Definition:** log1p\_exp.hpp:44

stan::prob::corr\_constrain

T corr\_constrain(const T x)

Return the result of transforming the specified scalar to have a valid correlation value between -1 a...

**Definition:** transform.hpp:952

stan::agrad::sin

fvar< T > sin(const fvar< T > &x)

**Definition:** sin.hpp:14

stan::prob::factor\_U

void factor\_U(const Eigen::Matrix< T, Eigen::Dynamic, Eigen::Dynamic > &U, Eigen::Array< T, Eigen::Dynamic, 1 > &CPCs)

This function is intended to make starting values, given a unit upper-triangular matrix U such that U...

**Definition:** transform.hpp:43

stan::prob::identity\_free

T identity\_free(const T y)

Returns the result of applying the inverse of the identity constraint transform to the input...

**Definition:** transform.hpp:427

stan::math::logit

boost::math::tools::promote\_args< T >::type logit(const T a)

Returns the logit function applied to the argument.

**Definition:** logit.hpp:44

stan::prob::simplex\_free

Eigen::Matrix< T, Eigen::Dynamic, 1 > simplex\_free(const Eigen::Matrix< T, Eigen::Dynamic, 1 > &x)

Return an unconstrained vector that when transformed produces the specified simplex.

**Definition:** transform.hpp:1188

stan::prob::cholesky\_factor\_constrain

Eigen::Matrix< T, Eigen::Dynamic, Eigen::Dynamic > cholesky\_factor\_constrain(const Eigen::Matrix< T, Eigen::Dynamic, 1 > &x, int M, int N)

Return the Cholesky factor of the specified size read from the specified vector.

**Definition:** transform.hpp:1412

stan::prob::cov\_matrix\_constrain\_lkj

Eigen::Matrix< T, Eigen::Dynamic, Eigen::Dynamic > cov\_matrix\_constrain\_lkj(const Eigen::Matrix< T, Eigen::Dynamic, 1 > &x, size\_t k)

Return the covariance matrix of the specified dimensionality derived from constraining the specified ...

**Definition:** transform.hpp:1910

multiply\_lower\_tri\_self\_transpose.hpp

stan::prob::prob\_free

T prob\_free(const T y)

Return the free scalar that when transformed to a probability produces the specified scalar...

**Definition:** transform.hpp:927

stan::prob::ub\_free

boost::math::tools::promote\_args< T, TU >::type ub\_free(const T y, const TU ub)

Return the free scalar that corresponds to the specified upper-bounded value with respect to the spec...

**Definition:** transform.hpp:668

stan::prob::ordered\_constrain

Eigen::Matrix< T, Eigen::Dynamic, 1 > ordered\_constrain(const Eigen::Matrix< T, Eigen::Dynamic, 1 > &x)

Return an increasing ordered vector derived from the specified free vector.

**Definition:** transform.hpp:1224

error\_handling.hpp

stan::math::check\_simplex

bool check\_simplex(const char \*function, const Eigen::Matrix< T\_prob, Eigen::Dynamic, 1 > &theta, const char \*name, T\_result \*result)

Return true if the specified vector is simplex.

**Definition:** check\_simplex.hpp:32

stan::prob::corr\_matrix\_free

Eigen::Matrix< T, Eigen::Dynamic, 1 > corr\_matrix\_free(const Eigen::Matrix< T, Eigen::Dynamic, Eigen::Dynamic > &y)

Return the vector of unconstrained partial correlations that define the specified correlation matrix ...

**Definition:** transform.hpp:1722

stan::prob::simplex\_constrain

Eigen::Matrix< T, Eigen::Dynamic, 1 > simplex\_constrain(const Eigen::Matrix< T, Eigen::Dynamic, 1 > &y)

Return the simplex corresponding to the specified free vector.

**Definition:** transform.hpp:1103

stan::agrad::square

fvar< T > square(const fvar< T > &x)

**Definition:** square.hpp:15

stan::math::inv\_logit

boost::math::tools::promote\_args< T >::type inv\_logit(const T a)

Returns the inverse logit function applied to the argument.

**Definition:** inv\_logit.hpp:52

stan::prob::read\_cov\_matrix

Eigen::Matrix< T, Eigen::Dynamic, Eigen::Dynamic > read\_cov\_matrix(const Eigen::Array< T, Eigen::Dynamic, 1 > &CPCs, const Eigen::Array< T, Eigen::Dynamic, 1 > &sds, T &log\_prob)

A generally worse alternative to call prior to evaluating the density of an elliptical distribution...

**Definition:** transform.hpp:300

matrix\_error\_handling.hpp

stan::prob::prob\_constrain

T prob\_constrain(const T x)

Return a probability value constrained to fall between 0 and 1 (inclusive) for the specified free sca...

**Definition:** transform.hpp:875

stan::agrad::sum

fvar< T > sum(const Eigen::Matrix< fvar< T >, R, C > &m)

**Definition:** sum.hpp:14

stan::math::check\_unit\_vector

bool check\_unit\_vector(const char \*function, const Eigen::Matrix< T\_prob, Eigen::Dynamic, 1 > &theta, const char \*name, T\_result \*result)

Return true if the specified vector is unit vector.

**Definition:** check\_unit\_vector.hpp:27

stan::agrad::inv\_logit

fvar< T > inv\_logit(const fvar< T > &x)

**Definition:** inv\_logit.hpp:15

stan::agrad::tanh

fvar< T > tanh(const fvar< T > &x)

**Definition:** tanh.hpp:14

stan::prob::cov\_matrix\_free\_lkj

Eigen::Matrix< T, Eigen::Dynamic, 1 > cov\_matrix\_free\_lkj(const Eigen::Matrix< T, Eigen::Dynamic, Eigen::Dynamic > &y)

Return the vector of unconstrained partial correlations and deviations that transform to the specifie...

**Definition:** transform.hpp:1983

stan::prob::make\_nu

const Eigen::Array< T, Eigen::Dynamic, 1 > make\_nu(const T eta, const size\_t K)

This function calculates the degrees of freedom for the t distribution that corresponds to the shape ...

**Definition:** transform.hpp:343

stan::prob::ub\_constrain

boost::math::tools::promote\_args< T, TU >::type ub\_constrain(const T x, const TU ub)

Return the upper-bounded value for the specified unconstrained scalar and upper bound.

**Definition:** transform.hpp:604

stan::math::index\_type

Primary template class for the metaprogram to compute the index type of a container.

**Definition:** index\_type.hpp:21

math.hpp

stan::agrad::size\_type

Eigen::Matrix< double, Eigen::Dynamic, Eigen::Dynamic >::Index size\_type

**Definition:** typedefs.hpp:14

stan::prob::corr\_free

T corr\_free(const T y)

Return the unconstrained scalar that when transformed to a valid correlation produces the specified v...

**Definition:** transform.hpp:995

stan::prob::lb\_constrain

T lb\_constrain(const T x, const TL lb)

Return the lower-bounded value for the specified unconstrained input and specified lower bound...

**Definition:** transform.hpp:520

stan::agrad::sqrt

fvar< T > sqrt(const fvar< T > &x)

**Definition:** sqrt.hpp:15

stan::math::check\_cholesky\_factor

bool check\_cholesky\_factor(const char \*function, const Eigen::Matrix< T\_y, Eigen::Dynamic, Eigen::Dynamic > &y, const char \*name, T\_result \*result)

Return true if the specified matrix is a valid Cholesky factor.

**Definition:** check\_cholesky\_factor.hpp:30

stan::prob::unit\_vector\_free

Eigen::Matrix< T, Eigen::Dynamic, 1 > unit\_vector\_free(const Eigen::Matrix< T, Eigen::Dynamic, 1 > &x)

**Definition:** transform.hpp:1067

stan::prob::cholesky\_corr\_free

Eigen::Matrix< T, Eigen::Dynamic, 1 > cholesky\_corr\_free(const Eigen::Matrix< T, Eigen::Dynamic, Eigen::Dynamic > &x)

**Definition:** transform.hpp:1592

index\_type.hpp

stan::prob::positive\_ordered\_constrain

Eigen::Matrix< T, Eigen::Dynamic, 1 > positive\_ordered\_constrain(const Eigen::Matrix< T, Eigen::Dynamic, 1 > &x)

Return an increasing positive ordered vector derived from the specified free vector.

**Definition:** transform.hpp:1317

stan::prob::unit\_vector\_constrain

Eigen::Matrix< T, Eigen::Dynamic, 1 > unit\_vector\_constrain(const Eigen::Matrix< T, Eigen::Dynamic, 1 > &y)

Return the unit length vector corresponding to the free vector y.

**Definition:** transform.hpp:1015

stan::prob::lub\_free

boost::math::tools::promote\_args< T, TL, TU >::type lub\_free(const T y, TL lb, TU ub)

Return the unconstrained scalar that transforms to the specified lower- and upper-bounded scalar give...

**Definition:** transform.hpp:845

stan::prob::cov\_matrix\_free

Eigen::Matrix< T, Eigen::Dynamic, 1 > cov\_matrix\_free(const Eigen::Matrix< T, Eigen::Dynamic, Eigen::Dynamic > &y)

The covariance matrix derived from the symmetric view of the lower-triangular view of the K by K spec...

**Definition:** transform.hpp:1863

stan::prob::read\_corr\_L

Eigen::Matrix< T, Eigen::Dynamic, Eigen::Dynamic > read\_corr\_L(const Eigen::Array< T, Eigen::Dynamic, 1 > &CPCs, const size\_t K)

Return the Cholesky factor of the correlation matrix of the specified dimensionality corresponding to...

**Definition:** transform.hpp:139

stan::agrad::atan2

fvar< T > atan2(const fvar< T > &x1, const fvar< T > &x2)

**Definition:** atan2.hpp:16

stan::agrad::atanh

fvar< T > atanh(const fvar< T > &x)

**Definition:** atanh.hpp:16

stan::math::check\_positive

bool check\_positive(const char \*function, const T\_y &y, const char \*name, T\_result \*result)

**Definition:** check\_positive.hpp:54

stan::prob::identity\_constrain

T identity\_constrain(T x)

Returns the result of applying the identity constraint transform to the input.

**Definition:** transform.hpp:391

stan::prob::cov\_matrix\_constrain

Eigen::Matrix< T, Eigen::Dynamic, Eigen::Dynamic > cov\_matrix\_constrain(const Eigen::Matrix< T, Eigen::Dynamic, 1 > &x, typename math::index\_type< Eigen::Matrix< T, Eigen::Dynamic, 1 > >::type K)

Return the symmetric, positive-definite matrix of dimensions K by K resulting from transforming the s...

**Definition:** transform.hpp:1769

stan::agrad::cos

fvar< T > cos(const fvar< T > &x)

**Definition:** cos.hpp:15

stan::agrad::logit

fvar< T > logit(const fvar< T > &x)

**Definition:** logit.hpp:17

stan::prob::cholesky\_factor\_free

Eigen::Matrix< T, Eigen::Dynamic, 1 > cholesky\_factor\_free(const Eigen::Matrix< T, Eigen::Dynamic, Eigen::Dynamic > &y)

Return the unconstrained vector of parameters correspdonding to the specified Cholesky factor...

**Definition:** transform.hpp:1485

stan::prob::corr\_matrix\_constrain

Eigen::Matrix< T, Eigen::Dynamic, Eigen::Dynamic > corr\_matrix\_constrain(const Eigen::Matrix< T, Eigen::Dynamic, 1 > &x, typename math::index\_type< Eigen::Matrix< T, Eigen::Dynamic, 1 > >::type k)

Return the correlation matrix of the specified dimensionality derived from the specified vector of un...

**Definition:** transform.hpp:1644

stan::math::sum

double sum(std::vector< double > &x)

**Definition:** sum.hpp:10

stan::math::check\_less\_or\_equal

bool check\_less\_or\_equal(const char \*function, const T\_y &y, const T\_high &high, const char \*name, T\_result \*result)

**Definition:** check\_less\_or\_equal.hpp:52

stan::math::e

double e()

Return the base of the natural logarithm.

**Definition:** constants.hpp:86

stan::prob::read\_corr\_matrix

Eigen::Matrix< T, Eigen::Dynamic, Eigen::Dynamic > read\_corr\_matrix(const Eigen::Array< T, Eigen::Dynamic, 1 > &CPCs, const size\_t K)

Return the correlation matrix of the specified dimensionality corresponding to the specified canonica...

**Definition:** transform.hpp:181

stan::prob::lub\_constrain

boost::math::tools::promote\_args< T, TL, TU >::type lub\_constrain(const T x, TL lb, TU ub)

Return the lower- and upper-bounded scalar derived by transforming the specified free scalar given th...

**Definition:** transform.hpp:710

stan::math::E

const double E

The base of the natural logarithm, .

**Definition:** constants.hpp:14

stan::agrad::multiply\_lower\_tri\_self\_transpose

Eigen::Matrix< fvar< T >, R, R > multiply\_lower\_tri\_self\_transpose(const Eigen::Matrix< fvar< T >, R, C > &m)

**Definition:** multiply\_lower\_tri\_self\_transpose.hpp:17

stan::math::check\_positive\_ordered

bool check\_positive\_ordered(const char \*function, const Eigen::Matrix< T\_y, Eigen::Dynamic, 1 > &y, const char \*name, T\_result \*result)

Return true if the specified vector contains only non-negative values and is sorted into increasing o...

**Definition:** check\_positive\_ordered.hpp:29

stan::prob::factor\_cov\_matrix

bool factor\_cov\_matrix(const Eigen::Matrix< T, Eigen::Dynamic, Eigen::Dynamic > &Sigma, Eigen::Array< T, Eigen::Dynamic, 1 > &CPCs, Eigen::Array< T, Eigen::Dynamic, 1 > &sds)

This function is intended to make starting values, given a covariance matrix Sigma.

**Definition:** transform.hpp:88

stan::prob::ordered\_free

Eigen::Matrix< T, Eigen::Dynamic, 1 > ordered\_free(const Eigen::Matrix< T, Eigen::Dynamic, 1 > &y)

Return the vector of unconstrained scalars that transform to the specified positive ordered vector...

**Definition:** transform.hpp:1285

stan::prob::positive\_free

T positive\_free(const T y)

Return the unconstrained value corresponding to the specified positive-constrained value...

**Definition:** transform.hpp:491

stan::prob::positive\_constrain

T positive\_constrain(const T x)

Return the positive value for the specified unconstrained input.

**Definition:** transform.hpp:446

stan::prob::read\_cov\_L

Eigen::Matrix< T, Eigen::Dynamic, Eigen::Dynamic > read\_cov\_L(const Eigen::Array< T, Eigen::Dynamic, 1 > &CPCs, const Eigen::Array< T, Eigen::Dynamic, 1 > &sds, T &log\_prob)

This is the function that should be called prior to evaluating the density of any elliptical distribu...

**Definition:** transform.hpp:280

stan::math::multiply\_lower\_tri\_self\_transpose

matrix\_d multiply\_lower\_tri\_self\_transpose(const matrix\_d &L)

Returns the result of multiplying the lower triangular portion of the input matrix by its own transpo...

**Definition:** multiply\_lower\_tri\_self\_transpose.hpp:18

stan::math::check\_less

bool check\_less(const char \*function, const T\_y &y, const T\_high &high, const char \*name, T\_result \*result)

**Definition:** check\_less.hpp:52

stan::prob::CONSTRAINT\_TOLERANCE

const double CONSTRAINT\_TOLERANCE

**Definition:** transform.hpp:32

stan::prob::cholesky\_corr\_constrain

Eigen::Matrix< T, Eigen::Dynamic, Eigen::Dynamic > cholesky\_corr\_constrain(const Eigen::Matrix< T, Eigen::Dynamic, 1 > &y, int K)

**Definition:** transform.hpp:1512

sum.hpp

stan::agrad::log

fvar< T > log(const fvar< T > &x)

**Definition:** log.hpp:15

stan::agrad::exp

fvar< T > exp(const fvar< T > &x)

**Definition:** exp.hpp:16

stan::math::check\_square

bool check\_square(const char \*function, const Eigen::Matrix< T\_y, Eigen::Dynamic, Eigen::Dynamic > &y, const char \*name, T\_result \*result)

Return true if the specified matrix is square.

**Definition:** check\_square.hpp:24

stan::math::check\_greater\_or\_equal

bool check\_greater\_or\_equal(const char \*function, const T\_y &y, const T\_low &low, const char \*name, T\_result \*result)

**Definition:** check\_greater\_or\_equal.hpp:58

stan::agrad::log1p

fvar< T > log1p(const fvar< T > &x)

**Definition:** log1p.hpp:16

stan::math::log1m

boost::math::tools::promote\_args< T >::type log1m(T x)

Return the natural logarithm of one minus the specified value.

**Definition:** log1m.hpp:40

stan::prob::lb\_free

boost::math::tools::promote\_args< T, TL >::type lb\_free(const T y, const TL lb)

Return the unconstrained value that produces the specified lower-bound constrained value...

**Definition:** transform.hpp:570

check\_square.hpp

matrix.hpp


---

[ Stan Home Page ]

*© 2011–2014,
Stan Development Team.*
